# Supplementary material for: The relationship between noise annoyance, emotional labor and burnout in operating-room nurses: A protocol for cross-sectional study
Source: PLoS One. 2025 Apr 29;20(4):e0321731. doi: 10.1371/journal.pone.0321731 (PMC12040210; doi:10.1371/journal.pone.0321731)
Supplement: Attachment — Submitted filename: Response to Reviewers.docx [file pone.0321731.s002.docx]

**Response Letter**

Dear editor and reviewer:

Thank you for handling our submission and offering us an opportunity of revision, as well as providing us with valuable comments concerning our manuscript entitled “The relationship of noise annoyance, emotional labor and burnout in operating room nurses: protocol for a cross-sectional study” (ID: PONE-D-23-14792). Those comments are helpful for revising and improving our paper, as well as the important guiding significance to our research. We have studied the comments carefully and have addressed all the requirements and comments, and detailed all the changed made to the manuscript which we hope meet with approval.

**Journal Requirements:**

**Journal Requirement 1**: Please ensure that your manuscript meets PLOS ONE's style requirements, including those for file naming.

**Authors’ response:** Thank you very much for your honest advice. We have contacted a professional organization to make changes according to PLOS ONE's style requirements, which we hope will meet with approval.

**Journal Requirement 2**: We note that you have indicated that there are restrictions to data sharing for this study. PLOS only allows data to be available upon request if there are legal or ethical restrictions on sharing data publicly.

**Authors’ response:** We are very sorry for any inconvenience caused to the journal. Since this study is a research protocol, it is currently in progress and the data collection has not yet been completed. However, we have uploaded the pilot survey data set of this study with a DOI of 10.6084/m9.figshare.25466992, as the pilot survey has been completed at this stage. And relevant data from this study will all be made available upon study completion. The following explanations have been added in the revised manuscript, which are replicated here for your easy reference:

***Data Availability Statement***

*The data underlying the pilot survey presented in the study are available from a DOI of 10.6084/m9.figshare.25466992. All subsequent relevant data from this study will be made available upon study completion. (Page 15, Line 314-317)*

**Journal Requirement 3**: Your ethics statement should only appear in the Methods section of your manuscript. If your ethics statement is written in any section besides the Methods, please move it to the Methods section and delete it from any other section. Please ensure that your ethics statement is included in your manuscript, as the ethics statement entered into the online submission form will not be published alongside your manuscript.

**Authors’ response:** Thank you very much for your practical advice, based on your request we have deleted the ethics statement written in another section besides the Methods.

**Reviewers' comments**

**Overall response to Reviewer:** Thank you very much for your positive response and kind suggestions on our work. We have endeavored to incorporate the feedback and have revised the manuscript accordingly. The detailed point-by-point responses are listed below. All changes made to the manuscript are marked in red so that they may be easily identified.

**Reviewers' comment 1:** The line: “In addition, noise …exposure to noise [6,7]” is a bit strange to me because how is noise annoyance accompanied by cognitive assessment?

**Authors’ response:** For the first comment, thank you very much for your suggestion, in response to which we have again reviewed and found that there was an error in the expression here. Our true intention was to express that noise annoyance can lead to impairments in cognitive assessment, and we have corrected this. We believe the added explanation help to address your concerns. The following sentence has been modified in the revised manuscript, which is replicated here for your easy reference:

*In addition, noise annoyance is often accompanied by negative emotional responses such as restlessness, distress, exhaustion, and cognitive assessment impairment, as well as a lack of control over exposure to noise [6,7]. (Page 3, Line 47-50)*

**Reviewers' comment 2:** It is stated that, compared to ward nurses OR nurses should provide patients greater compassionate care and emotional support. This needs a more in depth explanation and references.

**Authors’ response:** Thank you so much for your suggestion. We understand you are concerned about this statement, which is missing a more in-depth explanation and references. Regarding this concern, we have added more explanations in the revised manuscript as follows:

*For patients, being transferred from the ward to the operating room means they enter a relatively closed and unfamiliar environment, and be separated from their family members causes psychological discomforts to them. Due to preoperative restrictions on food and water, other factors such as poor rest at night before surgery also bring physical discomforts to them. In addition, surgical treatment contributes both physical and psychological traumas to patients to a certain extent, which increases patients' preoperative stress and anxiety. As a result, operating-room nurses need to provide more compassionate care and emotional support to patients [11]. (Page 3, Line 59-66)*

**Reviewers' comment 3:** Please describe more clearly when and how the data are collected. Additionally, also how participants are recruited (advertisement, via the wards, social media, etc.).

**Authors’ response:** We are grateful for your suggestion that we should make more detail about data collection. To illustrate this issue more clearly, we include measurement as a separate paragraph. And we have provided a more detailed description of the data collection and participant recruitment methods in the “Measurement” section. We believe the added explanation help to address your concerns. The following explanations have been added in the revised manuscript, which are replicated here for your easy reference:

*Between February 18 and March 17, 2023, a pilot survey was conducted on 30 nurses in OR of the Affiliated Hospital of ** Medical University by research assistants, and questionnaires were filled out on site. Based on the pilot test results, the content of the questionnaire was adjusted to form a final version. Subsequently, we started a formal investigation on March 18th, 2023. Officials from ** Medical University, along with the director of the Nursing Department of the Affiliated Hospital of ** Medical University contacted the head nurse in OR of the target hospitals, and research assistants explained the purpose and relevance of the study to them. The head nurse of the OR of the target hospital recruited nurses who meet the inclusion criteria. After obtaining respondents' verbal consent for data collection, they were issued an electronic questionnaire and an electronic informed consent form. In addition, nurses who completed the questionnaire were be paid 2 RMB for their time and effort. Data collection was scheduled to the end of July 2023, but the exact time depended on the progress of the research. (Page 10, Line 212-225)*

**Reviewers' comment 4:** Add (more) information about the reliability and validity of the used questionnaires and instruments (including references).

**Authors’ response:** We are grateful for your suggestion, and we understand that valid data comes from reliable questionnaires. Since the pilot survey of this study has been completed, we calculated the reliability of the questionnaires based on the results of the pilot survey. We believe the added explanation help to address your concerns. The following information have been added in the revised manuscript, which are replicated here for your easy reference:

*The noise annoyance level of the subjects will be measured by the descriptive rating scale recommended by …average score of 4 and above as high annoyance [36].* *The measure’s Cronbach’s alpha value was 0.947 in pilot survey of this study. (Page 8-9, Line 164-177)*

*The Emotional Labor Scale (ELS) will adopt the three-dimensional … in nurse groups. The questionnaire’s Cronbach's alpha value for the three dimensions are 0.711, 0.826 and 0.872, respectively [38], and 0.838, 0.858, and 0.713 respectively in pilot survey of this current study. (Page 9, Line 178-183)*

*This study will use the MBI-HSS (MBI Human Services Survey) scale … nursing population [39]. The Cronbach's alpha coefficients for each dimension of the questionnaire are between 0.800 and 0.850 in* *a Chinese study [39]**, and 0.950, 0.923, and 0.953 in pilot survey of our research. (Page 9-10, Line 191-195)*

**Reviewers' comment 5:** Please explain the sentence “… whether authorized personnel will be considered.”

**Authors’ response:** Thank you for your comment, and we are very sorry for any inconvenience this caused. “Whether authorized personnel” means an official public position in China. We have changed it to hospital tenure, a more international language. The specific modifications are as follows:

*… Work-related factors such as work experience as an OR nurse, professional title, annual income, and hospital tenure are also considered. (Page 8, Line 154-156)*

*Potential confounders as contributors to burnout…, satisfaction with salary and hospital tenure. (Page 13, Line 267)*

**Reviewers' comment 6:** The average hours of noise exposure per day are collected. However, is this by participant report? This might have a major recall bias. Moreover, when is sound regarded as ‘noise’?

**Authors’ response:** Thank you for your comment. This is one of the limitations of this study since its nature. First, due to the difficulty of working shifts, we are unable to measure the duration of noise exposure for each nurse, so this study chooses the form of participant self-report for data collection. Secondly, individuals have different perceptions and sensitivities to noise, which may cause bias in the noise annoyance and the duration of exposure to noise reported by research subjects [1, 2], but these variables are internal experiences that are best reported by individuals themselves. Consequently, we believe that self-reports are suitable for measuring such variables. And we will conduct further targeted investigation in the future. We hope this explanation helps to address your concerns. The following information have been added in the revised manuscript, which are replicated here for your easy reference:

*A limitation of this study is that … In addition, our use of self-reported measures can also be considered a study limitation, which may introduce a risk of social desirability bias. (Page 14, Line 295-296)*

**Reviewers' comment 7:** The authors ask the participants: “what personality traits do you think you belong to?” Is this based on a thorough valid and reliable personality test? If so provide this information. If not provide information why this question can replace a personality test.

**Authors’ response:** Thank you for your comment. This is also one of the limitations of this study. The purpose of this study is to explore the relationship between noise annoyance, emotional labor and burnout. The relationship between personality traits and burnout is not the focus of this investigation. In addition, three main scales were involved in this study, with a total of 41 items. If a standard scale is used to describe personality traits, there will be too many items, which will affect the quality of the data [3]. Because as far as we know, the comprehensively valid and reliable personality trait test scales in China have between 15 and 48 items [4, 5]. Therefore, we used this single item to collect data on participants' personality traits, described in demographic variables. In future exploration of the impact of personality traits on burnout in more detail, we will use more comprehensive and valid measurement scales. We hope this explanation helps to address your concerns. The following information have been added in the revised manuscript, which are replicated here for your easy reference:

*A limitation of this study is that personality traits of participants are not be measured using a thorough valid and reliable instrument since this is not the main objective of this study. In future exploration of the impact of personality traits on burnout in more detail, we will use more comprehensive and valid measurement scales. (Page 14, Line 289-292)*

**Reviewers' comment 8:** He sentence “the study will … nurse translation.” is unclear. Please rephrase.

**Authors’ response:** We are very grateful for your suggestion, and we are sorry for any inconvenience this caused. Since this sentence is indeed unclear, we have rephrased it. The following sentence has been modified in the revised manuscript, which are replicated here for your easy reference:

*This study will use the MBI-HSS (MBI Human Services Survey) scale translated and revised by scholars at the Hong Kong Polytechnic University for the nursing population [39]. (Page 9, Line 191-193)*

**Reviewers' comment 9:** The authors define a score in the MBI-HSS above a certain level as positive. Please provide references and/or a thorough explanation why the authors think this is a methodologically sound way to do this.

**Authors’ response:** We are very grateful for your suggestion, and we have provided references. The following information have been added in the revised manuscript, which are replicated here for your easy reference:

*We define that a score above or equal to 27 on the emotional exhaustion dimension, above or equal to 8 on the depersonalization dimension, or below or equal to 24 on the personal fulfillment dimension is positive [40]. Additionally, the scores of the three dimensions all lower than the threshold value are considered to be without burnout; the score of one dimension higher than the threshold value is viewed as mild burnout; the score of each of the two dimensions higher than the threshold value is viewed as moderate burnout; and the scores of the three dimensions higher than the critical value is viewed as severe burnout [40]. Besides, the total incidence rate of OR nurse burnout = (number of people with mild burnout + number of people with moderate burnout + number of people with severe burnout) / total number of people × 100% [41]. (Page 10, Line 199-209)*

**Reviewers' comment 10:** Sorry, but I can’t follow the sentence “Additionally, … total x 100%. Please make this more clear.

**Authors’ response:** We are very sorry for the inconvenience of reading this and thank you very much for your suggestion, we have amended the sentence as follows, which we hope will help you understand our research better.

*Besides, the total incidence rate of OR nurse burnout = (number of people with mild burnout + number of people with moderate burnout + number of people with severe burnout) / total number of people × 100%. (Page 10, Line 206-209)*

**Reviewers' comment 11:** In the calculation the mild, moderate and severe burnout are divided by ‘total’. What does this ‘total’ mean? Please explain.

**Authors’ response:** Thank you so much for your comment. The “total” refers to the “total number of people”. We are deeply sorry for the unclear expression. We hope this explanation helps to address your concerns.

**Reviewers' comment 12:** In the measurements and Bias section, is stated that the researchers will explain the purpose and significance of the survey. Shouldn’t this be the purpose and relevance?

**Authors’ response:** We are very grateful for your suggestion, and we have made the modification in our manuscript. The following sentence has been modified in the revised manuscript, which are replicated here for your easy reference:

*Between February 18 and March 17, 2023, a pilot survey was conducted … contacted the head nurse in OR of the target hospitals, and research assistants explained the purpose and relevance of the study to them. (Page 11, Line 219)*

**Reviewers' comment 13:** Nurses who completed the questionnaire will be paid for their time and effort. State how much money this is. Moreover, have the authors thoughts how the minimize the bias this might give?

**Authors’ response:** We are very grateful for your suggestion. The following explanation have been added in the revised manuscript, which are replicated here for your easy reference:

*Between February 18 and March 17, 2023, a pilot survey was conducted … In addition, nurses who completed the questionnaire were be paid 2 RMB for their time and effort. (Page 11, Line 222-223)*

Furthermore, you mentioned the possible bias this could introduce. In fact, we considered that a larger amount may prompt nurses to fill in the questionnaire in order to get the reward, so the reward amount we set was not high. Low reward cannot motivate mercenary people to fill in the questionnaire. This may reduce bias to some extent. We hope this explanation helps to address your concerns.

**Reviewers' comment 14:** What do the authors mean by: the baselines are basically close?

**Authors’ response:** Thank you for your comment, and we are very sorry for the lack of clarity. The baseline described in this study is basically close, which means that the subjects of this study come from the same region, have basically the same social culture, and the patients they face are basically similar. Moreover, the nurses recruited will all come from tertiary hospitals and have similar workloads. This makes the results comparable. We have modified this sentence, and the following explanation have been added in the revised manuscript, which are replicated here for your easy reference:

*We tried to control for non-response bias by … and all hospitals are tertiary hospitals. Since all the participants will come from tertiary hospitals in the same region, they have basically the same social culture and similar workloads. This makes the results comparable. (Page 12, Line 238-240)*

**Reviewers' comment 15:** In the statistical analysis is stated that data that has “obvious regularity and consistency” will be deleted. What does this mean? How is this addressed? And what methods are used for this check?

**Authors’ response:** Thank you so much for your comment. For “what methods are used for this check?”, we will review the returned questionnaires one by one to check whether the questionnaires are filled in effectively. If the data has obvious regularity or consistency (for example, if you select the same option in the entire questionnaire), the questionnaire will be considered invalid [6], and we will delete it. We have added this explanation and the reference in our revised manuscript as follows:

*We will review the returned questionnaires one by one to check whether the questionnaires are filled in effectively. If the data has obvious regularity or consistency (for example, if you select the same option in the entire questionnaire), the questionnaire will be considered invalid [42], and we will delete it. (Page 12, Line 243-246)*

**Reviewers' comment 16:** Please add information about the outcomes of the spearman and person correlations. When is it by example a weak or moderate correlation?

**Authors’ response:** We are very grateful for your suggestion, and the following explanation have been added in the revised manuscript, which are replicated here for your easy reference:

*Pearson correlation coefficient or Spearman correlation coefficient (according to the results of the normality test) will be used to explore the correlation between variables. For the correlation coefficient, 0 means there is no correlation between the two, -1 means negative correlation, and 1 means positive correlation. The closer it is to -1 or 1, the stronger the correlation [43]. (Page 13, Line 259-262)*

**Reviewers' comment 17:** In the potential confounders ‘whether authorized personnel’ is stated again. Please explain this.

**Authors’ response:** Thank you for your comment. Hospital tenure is a bit like that of a civil servant in China. Relatively speaking, contract nurses are more unstable, which to a certain extent becomes a potential confounding factor affecting burnout [7]. We hope this explanation helps to address your concerns.

**Reviewers' comment 18:** It is stated that participants will be interviewed. How will this be done? And why is this not described in the method section? Please add this to the method section.

**Authors’ response:** We apologies for any misunderstanding caused by our lack of clarity and thank you very much for your advice. We haven't made it clear here. This study will not conduct interviews with the research subjects. What we want to express here is that participants are investigated. And we made the modification in our manuscript as follows:

*Participants are investigated about their sociodemographic characteristics, noise irritation, emotional labor, and burnout. (Page 15, Line 299)*

**Reviewers' comment 19:** In addition, who are the trained research assistants? How are they trained, what is their professional background? Are they involved in the study? Please add this to the method section.

**Authors’ response:** Thank you for your comment. The trained research assistants in this study are Jiaqin Rao, Shasha Luo, and Qingjiao Guo, members of the research team. Their professional background is a master's degree in nursing, and the training content included the types, structures, question formulations, response types and survey methods of questionnaires by professional courses. In addition, they all participated in and contributed to this study, and the division of labor was detailed in our contribution statement. Furthermore, we added the explanation to the Methods section as follows:

*In this study, the research assistants were trained and qualified nursing master's students. The training content included the types, structures, question formulations, response types and survey methods of questionnaires by professional courses. (Page 11, Line 226-228)*

**Closing comment to Reviewer:** We hope the revised manuscript is now acceptable to you. If not, we are glad to receive any further feedback which we shall continue to apply our best effort to address. Once again, thank you very much for your comments and suggestions.

Thank you and best regards.

Yours sincerely,

Yizhi Zhang

References

1.	Abbasi M, Yazdanirad S, Zokaei M, et al. A Bayesian network model to predict the role of hospital noise, annoyance, and sensitivity in quality of patient care. *BMC Nurs.* 2022;21(1):243.

2.	Sung JH, Lee J, Jeong KS, et al. Influence of Transportation Noise and Noise Sensitivity on Annoyance: A Cross-Sectional Study in South Korea. *Int J Environ Res Public Health.* 2017;14(3):322.

3.	Burisch M. Approaches to personality inventory construction: A comparison of merits. *American psychologist.* 1984;39(3):214.

4.	Eysenck HJ, Eysenck SBG. *Manual of the Eysenck Personality Scales (EPS Adult): Comprising the EPQ-Revised (EPQ-R), EPQ-R Short Scale, Impulsiveness (IVE) Questionnaire.* Hodder & Stoughton; 1991.

5.	Zhang X, Wang MC, He L, et al. The development and psychometric evaluation of the Chinese Big Five Personality Inventory-15. *PLoS One.* 2019;14(8):e0221621.

6. Hongyan W, Hongzhen X, Yilan L, et al. Current status of knowledge，attitude and practice of humanistic care on nurses in 27 provinces and autonomous regions of China：a multicenter investigation. *Chinese Journal of Nursing.* 2023;58(19):2387-2394.

7. Guller U, Bühler L, Clavien PA. Statistics in medicine. Swiss Med Wkly. 2003 Oct 11;133(39-40):521. doi: 10.4414/smw.2003.10504. PMID: 14655051.
