# Supplementary material for: The relationship between noise annoyance, emotional labor and burnout in operating-room nurses: A protocol for cross-sectional study
Source: PLoS One. 2025 Apr 29;20(4):e0321731. doi: 10.1371/journal.pone.0321731 (PMC12040210; doi:10.1371/journal.pone.0321731)
Supplement: Attachment — Submitted filename: Response_to_Reviewers_auresp_2.docx [file pone.0321731.s003.docx]

**Response Letter**

Dear Editor and Reviewers:

Thank you for handling our submission and offering us an opportunity of revision, as well as providing us with valuable comments concerning our manuscript entitled “The relationship of noise annoyance, emotional labor and burnout in operating room nurses: protocol for a cross-sectional study” (ID: PONE-D-23-14792). Those comments are helpful for revising and improving our paper, as well as the important guiding significance to our research. We have studied the comments carefully and have addressed all the requirements and comments, and detailed all the changed made to the manuscript which we hope meet with approval.

**Reviewers' comments**

**Overall response to Reviewer:** Thank you very much for your positive response and kind suggestions on our work. We have endeavored to incorporate the feedback and have revised the manuscript accordingly. The detailed point-by-point responses are listed below.

**Reviewer #2**

**Comment 1:** The research topic of this manuscript is: The relationship of noise annoyance, emotional labor and burnout in operating room nurses. This manuscript contains some statements that are not relevant to the research topic. For example, surgical treatment contributes both physical and psychological traumas to patients to a certain extent, which increases patients' preoperative stress and anxiety. operating-room nurses need to provide more compassionate care and emotional support to patients [11]. Then, the author mentioned emotional labor. The context is incoherent. There are logical problems with the content of this manuscript. The content requires major revision.

**Authors’ response:** Thank you very much for your comment. Your suggestion have truly helped us improve the quality of the paper. Based on your recommendation, we have removed irrelevant statements and made major revisions to the content. We believe the revisions help to address your concerns. The following sentences have been modified in the revised manuscript, and are replicated here for your easy reference:

***Introduction***

*Burnout represents a significant challenge to the well-being of nurses and their professional environment [1]**. Defined as a prolonged response to chronic interpersonal and emotional stressors in the workplace, burnout is a complex occupational psychosocial syndrome [2]. In mainland China, the prevalence of burnout among registered nurses is alarmingly high, reaching up to 50% [3]. Operating Room (OR) nurses, due to the high-intensity, high-risk, and overload of their work, are particularly vulnerable to burnout [4]. A study conducted by Ren Lanzhu reported that 78.77% of OR nurses experience varying degrees of burnout [5]. The implications of burnout extend beyond individual well-being, affecting nurses’ physical and mental health, increasing turnover intentions, and negatively impacting care quality, patient safety, and job satisfaction [6]. Moreover, research indicates that burnout among nurses correlates with higher patient mortality rates and increased hospital-acquired infections [7]. Therefore, exploring the factors contributing to burnout in OR nurses and identifying effective intervention strategies are crucial for improving their mental health.*

*Noise annoyance (NA) is defined as the adverse emotional response of individuals or groups exposed to noisy environments. It manifests through feelings of distraction, discomfort, irritation, and anxiety, serving as one of the most significant psychological impacts of noise [8,9]。Noise annoyance is widely regarded as a key indicator of adverse subjective reactions to noise. OR nurses are likely to experience heightened levels of noise annoyance due to their work environment. A study revealed that 95.2% of anesthesiologists working in ORs reported annoyance caused by noise, with 14.5% experiencing high levels of noise annoyance [10]. Furthermore, burnout is closely linked to negative emotional states [11]. Noise annoyance, an often overlooked negative emotion [12], may be significantly associated with burnout. However, the precise relationship between noise annoyance and burnout remains underexplored, warranting further investigation.*

*Emotional labor (EL) refers to the process by which individuals regulate their emotions to align with organizational expectations, resulting in specific facial expressions and behavioral displays required by their roles [13]. OR nurses frequently engage in emotional labor to provide patient care [14]. Bartram confirmed that emotional labor is a significant positive predictor of burnout[15]. And negative emotions are one of the factors influencing emotional labor [16]. Since noise annoyance is a form of negative emotion [12], the relationship between noise annoyance and emotional labor in OR nurses has yet to be explored.*

*While the relationship between emotional labor and burnout appears well-established, the link between noise annoyance and burnout remains unclear, and the role of emotional labor in the connection between noise annoyance and burnout is still unknown.* *Therefore, the objectives of this study are: (1)* *to investigate the independent correlation between noise annoyance and burnout among OR nurses; (2) to explore the relationship between noise annoyance, emotional labor, and burnout.*

***Research hypothesis***

*As early as 1988, Topf identified noise pollution as a significant stressor in the nursing work environment, with noise-induced stress serving as a predictive factor for burnout among clinical nurses [17]. Noise annoyance, as a specific form of psychological stress [18], may influence the onset of burnout, particularly given the well-established link between stress and burnout [19]. In addition, a survey of 2,000 nurses in Taiwan examining burnout identified feelings of tension, irritability, sadness, inferiority, and sleep disturbances as the most significant predictors of burnout [20]. Compared to factors such as job satisfaction, job engagement, and work environment, psychological factors were found to have the most substantial correlation with emotional exhaustion* *[13,14]. Negative emotions have also been shown to correlate positively with burnout[11]. From a psychological perspective, noise annoyance in OR nurses represents a form of negative emotion[18], which may, in turn, contribute to burnout.*

*The Conservation of Resources theory posits that the persistent threat of resource loss can lead to burnout, ultimately impacting patient safety [19]. This theory is grounded in the premise that individuals strive to acquire, maintain, and protect resources they value, providing a useful framework for understanding burnout. It highlights the psychological stress experienced by workers in human services, including healthcare and education, as a result of interpersonal demands [21,22]. For OR nurses, exposure to noise annoyance can deplete emotional resources, and if these resources are not replenished in a timely manner, further depletion may lead to burnout.*

*Grandey identified several factors influencing emotional labor, including emotional intelligence, emotional expression, emotional disposition, and emotional events (both positive and negative) [23]. And negative emotions are significantly correlated with emotional labor [16], which is considered a key predictor of burnout [24]. The Self-Regulation Theory refers to the process by which individuals control or alter their thoughts, emotions, impulses, and behaviors to achieve specific goals [25]. In the context of emotional labor, OR nurses adjust and display appropriate emotions to meet work requirements or objectives, exemplifying a typical self-regulation process. Before engaging in emotional labor for patients, OR nurses may already be experiencing varying degrees of noise annoyance. The higher the level of noise annoyance, the more pronounced the emotional dysregulation, which increases the likelihood of emotional exhaustion and ultimately leads to burnout. Furthermore, when OR nurses engage in emotional labor, noise annoyance forces them to exert more self-regulation, thereby increasing the demand for emotional resources. When emotional demands and resources are significantly out of balance, burnout is likely to accelerate.*

*Based on these considerations, this study proposes the following hypotheses:*

*a) There is a positive correlation between noise annoyance and burnout among OR nurses.*

*b) There is a positive correlation between noise annoyance and emotional labor among OR nurses.*

*c) There is a positive correlation between emotional labor and burnout among OR nurses.*

*d) Emotional labor mediates the relationship between noise annoyance and burnout among OR nurses.*

*The hypothesized model for this study is illustrated in Fig 1. (Page 2-7, Line 38-129)*

**Comment 2:** In addition, the authors set four predictor variables when estimating the minimum sample size. But according to the authors' research structure there are only two predictor variables. What are the other two variables?

**Authors’ response:** Thank you for highlighting the discrepancy regarding the number of predictor variables used in our sample size estimation. We appreciate the opportunity to clarify this aspect of our study. In our research, while we primarily focus on two main predictor constructs—noise annoyance and emotional labor—the construct of emotional labor is further subdivided into three distinct dimensions: surface acting, deep acting, and natural emotional expression. Each of these dimensions represents a unique aspect of emotional labor and is treated as a separate predictor variable in our multiple regression analysis. Therefore, in total, we have four predictor variables: Noise Annoyance, Surface Acting, Deep Acting, and Natural Emotional Expression. This approach allows us to explore the nuanced effects of each dimension of emotional labor on burnout, providing a more comprehensive understanding of the factors contributing to burnout among operating room nurses. We hope the explanation helps to address your concerns. And we have made the revisions as follows:

*The required sample size was calculated using G*Power 3.1.9.7 software [26] for multiple linear regression analysis, with* ***four predictor variables: noise annoyance, surface acting, deep acting, and natural emotional expression****, and burnout as the outcome variable. While emotional labor is our primary construct of interest, it is operationalized through three distinct dimensions—surface acting, deep acting, and natural emotional expression—each serving as an individual predictor. This differentiation allows for a more nuanced examination of how each aspect of emotional labor contributes to burnout among OR nurses.(Page 7-8, Line 147-154)*

**Comment 3:** According to the research hypothesis. This manuscript does not require independent samples t-test and variance analysis. Why should the author consider control variables? What is the theoretical basis? It is recommended that the author seek assistance from statistical experts. From the manuscript's content, I am concerned that the statistical analysis of this manuscript is wrong.

**Authors’ response:** Thank you for your valuable comment. We appreciate the opportunity to clarify the rationale behind our methodological approach.

**Justification for Including Control Variables:**

Our primary objective is to investigate the independent association between noise annoyance and burnout among operating room nurses. Burnout is a complex, multifactorial phenomenon influenced by various personal and professional factors [1, 2]. To accurately assess the unique effect of noise annoyance on burnout, it is essential to control for potential confounding variables that may also impact burnout levels.

**Theoretical Basis:**

The inclusion of control variables is grounded in the Conservation of Resources Theory Model which mentioned in the INTRODUCTION. According to this model, burnout develops when job demands are high and resources are insufficient. Personal characteristics (e.g., age, marital status, education level, personality traits) and job-related factors (e.g., work experience, professional title, income, job satisfaction) can influence both the perception of noise annoyance and the experience of burnout [1, 2].

By controlling for these variables, we aim to isolate the specific effect of noise annoyance on burnout, ensuring that our findings are not confounded by other factors known to influence burnout.

**Use of Independent Samples t-test and ANOVA (Univariate Analysis):**

Prior to conducting multiple regression analysis, we perform univariate analyses (independent samples t-tests and one-way ANOVA) to identify potential confounders. This step helps us determine which demographic and professional variables are significantly associated with burnout and should be included as control variables in the regression model.

We hope the explanations help to address your concerns.

**Comment 4:** Finally, the research structure of this manuscript is logically correct. However, the textual narrative lacks logic. This manuscript still requires major revision.

**Authors’ response:** Thank you so much for your comment. Your suggestion have truly helped us improve the quality of the paper. We have made major revisions to the manuscript and enhanced the logical flow of the text. Due to space limitations, we kindly ask the reviewer to refer back to the manuscript. Thank you once again.

**Reviewer #3**

**Comment 1:**

Line 133: The exact name is to be stated if the manuscript is accepted for publication.
Line 143: For the sample size calculation, the outcome variable needs to be specified. For G*Power, indicate the test family, the statistical test, and the type of power analysis used.
Line 204-206: Use brackets to present the formula, ensuring that 100% is clearly separated from the denominator.
Line 214: Replace the word ‘effectively’ with a more appropriate alternative.
Line 244: Proper citation of the SPSS including the publisher's name is to be stated.
Line 253: Replace the word ‘parametric’ with ‘non-parametric’.
Line 256: Specify that the p-value is based on a two-sided test.
Line 266-268: Clearly indicate whether the analysis will be conducted using the SPSS PROCESS macro or another method.
Line 270: The word ‘significant’ is to be replaced with ‘statistically significant’.
Line 314: Provide the access weblink.
Line 318: The sentence is unclear and needs revision.
**Authors’ response:** Thank you very much for your valuable comments. Your suggestions have truly helped us improve the quality of the manuscript. We have made revisions based on your comments and have replicated here for your easy reference:

*The* ***test family used was F-tests, and the statistical test employed was linear multiple regression (Fixed model, R² deviation from zero). A priori: Compute required sample size - given α, power, and effect size, was used for the type of power analysis. Assuming a medium effect size (f² = 0.15), an alpha level of 0.05, and a desired power of 0.95,*** *the minimum required sample size was determined to be 129 participants. To account for a potential 20% dropout rate, the final minimum sample size was calculated to be 155. This sample size ensures sufficient power to detect significant relationships within our multiple regression model [27].(Page 8, Line 155-162)*

*Besides, the total incidence rate of OR nurse burnout =****[*** *(number of people with mild burnout + number of people with moderate burnout + number of people with severe burnout) / total number of people****]*** *× 100%. (Page 11, Line 218-221)*

*We will review the returned questionnaires one by one to check whether they are filled in* ***completely****. (Page 13, Line 256-257)*

*IBM SPSS Statistics 26.0* ***(IBM Corp., 2019)*** *will be used for statistical analysis*. *(Page 13, Line 260)*

*If the variance is homogeneous, we will use independent sample student’s t-test and one-way ANOVA; if not, we will use* ***non-parametric tests*** *(Wilcoxon rank sum test and Kruskal-Wallis H test). (Page 13, Line 267-269)*

*A p-value of 0.05 or lower will be regarded as* **statistically *significant****,* ***based on a two-sided test.*** *(Page 15, Line 301-302)*

*We will use* ***Model 4 (PROCESS macro)*** *in SPSS 26.0 to examine the mediation effect of emotional labor on the relationship between noise annoyance and burnout among OR nurses [40].(Page 15, Line 296-298)*

*The data underlying the pilot survey presented in this study are available at* ***https://figshare.com/articles/dataset/____/25466992?file=45245002****, with the DOI 10.6084/m9.figshare.25466992. (Page 17, Line 340-344)*

***The authors did not receive any external funding to support this work.****(Page 17, Line 347)*

**Comment 2:** Line 303: Why was PLOS mentioned here? Does PLOS play a role in this study? Line 300 and 303: These sentences need revision.

**Authors’ response:** Thank you for your comment. We have reviewed this sentence and agree that it was not appropriate, so we have removed the statement.

**Comment 3:** The fulfillment of multiple regression assumptions should be stated. If there are non-linear relationships between variables, the results may not accurately reflect the true mediation effect. Line 260-269: More details could be provided for this section.

**Authors’ response:** We fully understand your concerns. Therefore, we have clarified the assumptions for conducting multiple linear regression and provided solutions for handling potential nonlinear relationships between the variables. We hope this addresses your concerns. The added details are as follows:

*We will carry out linear multiple regression analysis to control for confounders and determine whether noise annoyance and emotional labor are contributors to burnout. Before performing multiple regression analyses, we will examine scatterplots of each predictor variable against the burnout score to check for linear relationships. The normality of residuals will be tested using the Shapiro-Wilk test and visualized with Q-Q plots. And we will evaluate homoscedasticity by plotting residuals versus predicted values. The Durbin-Watson statistic will be used to test for autocorrelation of residuals. In case non-linear relationships are identified, we will consider applying transformations to the predictor variables or incorporating polynomial or interaction terms into the regression model to better capture the nature of the relationships. (Page 14, Line 280-290)*

**Comment 4:** When controlling for multiple confounders, there is a risk of multicollinearity, which can inflate standard errors and make it difficult to assess the unique contribution of each variable. This needs to be addressed. Line 260-269: More details could be provided for this section.

**Authors’ response:** Thank you very much for your comment. We have added statements in the manuscript discussing how to address the risk of multicollinearity arising from multiple confounders. The following information have been added in the revised manuscript, which are replicated here for your easy reference:

*To address the risk of multicollinearity arising from multiple confounders, we will calculate Variance Inflation Factors (VIF) and tolerance levels for each predictor variable. A VIF value exceeding 5 or a tolerance value below 0.2 will indicate significant multicollinearity [39]. If multicollinearity is detected, we will consider removing highly correlated variables. (Page 14, Line 291-295)*

**Comment 5:** Single-item measure is generally inadequate for capturing the personality traits and could lead to inaccurate/incomplete assessments even if it is not the primary focus/objective of the study.

**Authors’ response:** This is indeed a limitation of the study, and we have reiterated it in the limitations section. As the study is still ongoing, we are unable to modify this part at the moment. In future research, we will use a scale for the personality traits evaluation.

**Comment 6:** References: Some references do not conform to the journal’s format and the references is to be thoroughly checked.

**Authors’ response:** We are very grateful for your suggestion, and we have checked all the references. The following references have been modified in the revised manuscript, and are replicated here for your easy reference:

***References***

*1.	Sucapuca C, Morales-García WC, Saintila J. Work-related factors associated with burnout among Peruvian nurses. Journal of Primary Care & Community Health. 2022;13:21501319221127085.*

*2.	Leiter MP, Maslach C. Areas of worklife: A structured approach to organizational predictors of job burnout. Emotional and physiological processes and positive intervention strategies: Emerald Group Publishing Limited; 2003. p. 91-134.*

*3.	Zhang W, Miao R, Tang J, Su Q, Aung LHH, Pi H, et al. Burnout in nurses working in China: A national questionnaire survey. International journal of nursing practice. 2021;27(6):e12908.*

*4.	Xu Xiaobing, Ye L. Research progress on occupational burnout among operating room nurses. Qilu Nursing Journal. 2023;29(8):145-147.*

*5.	Ren Lanzhu, Yang Peng, Zhang Minli, Yanyan L. Study on the correlation between job burnout and sleep quality of operating room nurses in a grade-A tertiary hospital in Guangzhou. Psychology Monthly. 2023;18(23):30-33, 67. doi: 10.19738/j.cnki.psy.2023.23.007.*

*6.	Rathert C, Williams ES, Linhart H. Evidence for the Quadruple Aim: A*

*Systematic Review of the Literature on Physician Burnout and Patient Outcomes. Med Care. 2018;56(12):976-984. Epub 20181020. doi: 10.1097/mlr.0000000000000999. PubMed PMID: 30339573.*

*7.	Parada ME, Moreno B R, Mejías M Z, Rivas F A, Rivas F F, Cerrada S J, et al. Job satisfaction and burnout syndrome in the nursing staff of the Instituto Autónomo Hospital Universitario Los Andes, Mérida, Venezuela, 2005. Revista Facultad Nacional De Salud Pública. 2005;23(1):33-45.*

*8.	WHO. Health and Sustainable Development. Noise: World Health Organization; 2020-01-28 [cited 2024 March 20]. Available from: <http://www.who.int/sustainable-development/transport/health-risks/noise/en/>.*

*9.	WHO Regional Office for Europe J. Burden of disease from environmental noise: Quantification of healthy life years lost in Europe Copenhagen: WHO Regional Office for Europe; 2016 [cited 2024 March 20]. Available from: <http://www.who.int/quantifying_ehimpacts/publications/e94888/en/>.*

*10.	Wang Xiaoxiao, Wei Bin, Liu Xiaoli, Yiming Z. Investigation of noise annoyance among the exposed anesthesiologists in a hospital. Chinese journal of industrial hygiene and occupational diseases. 2021;39(10):775-778.(10). doi: 10.3760/cma.j.cn121094-20200528-00296.*

*11.	Yao Yong, Ni Lei, Cao Hui, Gao Yifang, Wu Jing, zhen W. Investigation on the emotional state and related factors of workers in a manufacturing plant in Wuhan. Journal of Public Health and Preventive Medicine. 2013;24(4):81-83.*

*12.	Stallen PJ. A theoretical framework for environmental noise annoyance. Noise Health. 1999;1(3):69-80. Epub 20030412. PubMed PMID: 12689501.*

*13.	Lu Shan, Xiaoling S. Research progress on intervention management of emotional labor in nurses. Chinese Journal of Nursing. 2021;56(1):137-142.*

*14.	Chen C. Study on the correlation between emotional labor and psychological resilience of operating room nurses in tertiary hospitals in Changchun City . Thesis, The University of Jilin. 2018. Available from: https://next.cnki.net/middle/abstract?v=W694F5cljyCSJNc_vasdvDL1oSLv21i9L7tByXZBu2PuaGqBxJpmiC_kmUbD4iKxsP8zt1baWawU3-WzA1JJ8kqBGUic4_6rDZf0oLG-4TXKT5Un9yfsWz4da9WluXCmmUnnBd6lfNlYKkX5b0AM5AtYeqcDVNkZWsZWzAAAeTkiuVbeVer-UFc7Hc8NaaJ3BY-nmkLtpLg=&uniplatform=NZKPT&language=CHS&scence=null*

*15.	Bartram T, Casimir G, Djurkovic N, Leggat SG, Stanton P. Do perceived*

*high performance work systems influence the relationship between emotional labour, burnout and intention to leave? A study of Australian nurses. Journal of Advanced Nursing. 2012;68(7):1567-1578.*

*16.	Qiaoyang B. The influencing factors of emotional labor and its relationship with employees' mental health.Thesis, The University of Jilin. 2006. Available from: https://next.cnki.net/middle/abstract?v=W694F5cljyDpdAtPwHqJXiSnVMT4n9r0BbqZXZ85cHx5QO9oDk-vMrZ8kw0NZindpcZwDR7Nt8bekYjAalJbhTAIz-iJLjWSyTjkCaHUPK110bz4_YwtK6iHxZVOhKGeDtIcfmibLVQV-qZVWvt_-lf4wEFiEUOjB7lnMGzwt_LxI-19Npuv29NNu2keUV0LUbiuOPz1aeo=&uniplatform=NZKPT&language=CHS&scence=null.*

*17.	Topf M, Dillon E. Noise-induced stress as a predictor of burnout in critical care nurses. Heart & lung: the journal of critical care. 1988;17(5):567-574.*

*18.	Stallen PJM. A theoretical framework for environmental noise annoyance. Noise and Health. 1999;1(3):69-79.*

*19.	Liao Huahua, Huang Lei, Bin H. Conservation of resources theory in the organizational behavior context:*

*Theoretical evolution and challenges. Advances in Psychological Science. 2022;30(2):449-463.*

*20.	Lee H-F, Yen M, Fetzer S, Chien TW. Predictors of burnout among nurses in Taiwan. Community mental health journal. 2015;51:733-737.*

*21.	Maslach C, Leiter MP. New insights into burnout and health care: Strategies for improving civility and alleviating burnout. Medical teacher. 2017;39(2):160-163.*

*22.	Freudenberger HJ. Staff burn-out. Journal of social issues. 1974;30(1):159-165.*

*23.	Grandey AA. Emotion regulation in the workplace: a new way to conceptualize emotional labor. J Occup Health Psychol. 2000;5(1):95-110. Epub 20000205. doi: 10.1037//1076-8998.5.1.95. PubMed PMID: 10658889.*

*24.	Smith P. Recruit and retain. The emotional labour of nursing. Nurs Times. 1988;84(44):50-51. Epub 19881102. PubMed PMID: 3205760.*

*25.	Bandura A. Social cognitive theory of self-regulation. Organizational behavior and human decision processes. 1991;50(2):248-287.*

*26.	Faul F, Erdfelder E, Buchner A, Lang AG. Statistical power analyses using G*Power 3.1: tests for correlation and regression analyses. Behav Res Methods. 2009;41(4):1149-1160. doi: 10.3758/brm.41.4.1149. PubMed PMID: 19897823.*

*27.	Cohen J. Statistical power analysis for the behavioral sciences. New York: Routledge imprint; 2013.*

*28.	Eysenck HJ, Eysenck SBG.* *Manual of the Eysenck Personality Scales (EPS adult), comprising the EPQ-revised ((EPQ-R)(including addiction and criminality scales) EPQ-R short scale impulsiveness (IVE) questionnaire (impulsiveness/venturesomeness/empathy). Hodder and Stoughton; 1991.*

*29.	University Z, University N, Technology HUoSa, Sciences SAoE. Acoustics—assessment of noise annoyance by means of social and socio-acoustics surveys. In: Vol GB/Z 21233-2007: General administration of quality supervision, inspection and quarantine of the people's republic of China. Administration of China; 2007. p. 16.*

*30.	Standardization I. Acoustics—Assessment of Noise Annoyance by Means of Social and Socio-Acoustic Surveys. ISO/TS. 2003;15666.*

*31.	AORN.* *AORN Position Statement on Managing Distractions and Noise During Perioperative Patient Care. Aorn Journal. 2020;111(6):675-680. doi: 10.1002/aorn.13064. PubMed PMID: WOS:000536490600016.*

*32.	Grandey AA. When "the show must go on": Surface acting and deep acting as determinants of emotional exhaustion and peer-rated service delivery. Academy of Management Journal. 2003;46(1):86-96. doi: 10.5465/30040678. PubMed PMID: WOS:000181209500007.*

*33.	Lou Hong, Sun Qingling, Lihui G. Effect of emotional labor on professional burnout of nurses. Chinese Journal of Nursing. 2008;(11):969-971.*

*34.	Sukun C. Clinical Psychological Nursing Instruction. Peking: Science and Technology Literature Publishing House; 2001: 207-218 p.*

*35.	Ye Zhihong, Luo Hong, Anli J. Diagnostic standard and norms of Maslach Burnout Inventory for nurses in Hangzhou. Chinese Journal of Nursing. 2008;(3):207-209.*

*36.	Wang Ying, Sha Liyan, Yang L. The influence of working environment alarm fatigue and emotional labor on Intensive Care Unit nurses' job burnout. Chinese Nursing Management. 2022;22(9):1405-1410.*

*37.	Li Lia, Zhao Lifen, She Dan, Xiong Zhuangzhi, Pei X. Effects of performance pressure and psychological capital on job burnout of nurses in emergency department of five tertiary hospitals in Wuhan City. Occupation and Health. 2022;38(20):2747-2752. doi: 10.13329/j.cnki.zyyjk.2022.0600.*

*38.	Dalton ER. STATISTICS IN MEDICINE - COLTON,T. International Journal of Epidemiology. 1975;4(4):348-349. doi: 10.1093/ije/4.4.348-b. PubMed PMID: WOS:A1975BA69800021.*

*39.	Kutner MH, Nachtsheim CJ, Neter J, Li W. Applied linear statistical models: McGraw-hill; 2005.*

*40.	Preacher KJ, Hayes AF. SPSS and SAS procedures for estimating indirect effects in simple mediation models. Behavior research methods, instruments, & computers. 2004;36:717-731.*

*41.	MacKinnon DP, Lockwood CM, Williams J. Confidence limits for the indirect effect: Distribution of the product and resampling methods. Multivariate Behavioral Research. 2004;39(1):99-128. doi: 10.1207/s15327906mbr3901_4. PubMed PMID: WOS:000222172500004.*

*42.	Preacher KJ, Hayes AF. Asymptotic and resampling strategies for assessing and comparing indirect effects in multiple mediator models. Behavior Research Methods. 2008;40(3):879-891. doi: 10.3758/brm.40.3.879. PubMed PMID: WOS:000257991700027.*

*(Page 17-20, Line 352-487)*

**Reviewer #4**

**Comment 1:** Line 144-146 Sample size: how many nurses are anticipated to have burn out? Is this the 5%. Will logistic regression be used? This needs to be understandable to which regression will be done, for which sort of outcome and predictor.

**Authors’ response:** Burnout will be assessed using the Maslach Burnout Inventory (MBI), which provides a continuous total score representing the level of burnout. Previous studies have reported varying levels of burnout among nurses, with scores indicating moderate to high levels. Therefore, we anticipate a range of burnout scores rather than a specific percentage of nurses experiencing burnout. And we have added a statement in the manuscript as follows:

*Since burnout is measured on a continuous scale, we consider that a higher score reflects more severe symptoms of burnout [37]. (Page 11, Line 221-222)*

**Comment 2:** The stats analysis section reads as if it may be linear regression if normally distributed outcome variable of burnout score. State this clearly in the analysis section and the sample size (the method to assess the primary outcome should be used in the sample size justification.

**Authors’ response:** Thank you for your helpful comment. We appreciate your suggestion and would like to clarify the statistical approach and sample size justification. We have amended the sentence as follows：

*The required sample size was calculated using G*Power 3.1.9.7 software [26] for multiple linear regression analysis,* ***with four predictor variables: noise annoyance, surface acting, deep acting, and natural emotional expression, and burnout as the outcome variable.*** *While emotional labor is our primary construct of interest, it is operationalized through three distinct dimensions—surface acting, deep acting, and natural emotional expression—each serving as an individual predictor. This differentiation allows for a more nuanced examination of how each aspect of emotional labor contributes to burnout among OR nurses.*

*The test family used was F-tests, and the statistical test employed was* ***linear multiple regression (Fixed model, R² deviation from zero)****. A priori: Compute required sample size - given α, power, and effect size, was used for the type of power analysis. Assuming a medium effect size (f² = 0.15), an alpha level of 0.05, and a desired power of 0.95, the minimum required sample size was determined to be 129 participants. To account for a potential 20% dropout rate, the final minimum sample size was calculated to be 155. This sample size ensures sufficient power to detect significant relationships within our multiple regression model [27]. (Page 8, Line 147-162)*

**References**

1.	Maslach C, Leiter MP. Understanding the burnout experience: recent research and its implications for psychiatry. World Psychiatry. 2016;15(2):103-111. doi: 10.1002/wps.20311. PubMed PMID: WOS:000379302100004.

2.	O'Sullivan S. The burnout companion to study and practice: a critical analysis. Ergonomics. 2021;64(11):1507. doi: 10.1080/00140139.2021.1959733. PubMed PMID: WOS:000679643400001.
